# Multi-attention bidirectional contrastive learning method for unpaired image-to-image translation

**DOI:** 10.1371/journal.pone.0301580

**Published:** 2024-04-16

**Authors:** Benchen Yang, Xuzhao Liu, Yize Li, Haibo Jin, Yetian Qu

**Affiliations:** Software College, Liaoning Technical University, Huludao, China; Nanchang University, CHINA

## Abstract

Unpaired image-to-image translation (I2IT) involves establishing an effective mapping between the source and target domains to enable cross-domain image transformation. Previous contrastive learning methods inadequately accounted for the variations in features between two domains and the interrelatedness of elements within the features. Consequently, this can result in challenges encompassing model instability and the blurring of image edge features. To this end, we propose a multi-attention bidirectional contrastive learning method for unpaired I2IT, referred to as MabCUT. We design separate embedding blocks for each domain based on depthwise separable convolutions and train them simultaneously from both the source and target domains. Then we utilize a pixel-level multi-attention extractor to query images from embedding blocks in order to select feature blocks with crucial information, thus preserving essential features from the source domain. To enhance the feature representation capability of the model, we incorporate depthwise separable convolutions for the generator. We conducted comprehensive evaluations using three datasets, demonstrating that our approach enhances the quality of unpaired I2IT while avoiding the issue of mode collapse-related image blurring.

## Introduction

In recent years, image-to-image translation (I2IT) has been widely used in a number of domains, including style transfer, image restoration, dehazing, super-resolution reconstruction, and more. Achieving precise transformations between the original area *S* and the goal area *T* is the aim of I2IT. This is done while ensuring the preservation of the fundamental content from the source domain in the transformation. One common task in the field of style transfer involves converting horse images into zebra images. The objective of this transfer is to convert an image of a horse to a zebra while preserving the original image’s background and structures.

CycleGAN [1], 1D Cycle-GAN [2], and AttenCGAN [3] address unique challenges by incorporating the concept of combining Generative Adversarial Networks (GAN) with cycle consistency. These approaches aim to constrain and improve the resemblance between artificially generated images and actual images. However, cycle consistency [1] can lead to distorted generated images. In order to generate more vibrant images, CUT [4], RRUIT [5], and SRC [6] attempt to reduce the constraints of cycle consistency [1]. CUT [4] introduces the method of block contrastive learning. With the goal to efficiently use the properties of the generator’s input and output domains for contrastive learning, this strategy suggests maximising the mutual information between input and output blocks. By imposing constraints on the features from the same position using the encoder, it ensures the preservation of important content in the source domain, thus leading to an improvement in the quality of image translation.

Nevertheless, CUT [4] lacks the capability to differentiate the effectiveness of negative samples, leading to a high level of randomness in the translation process. Qs-Attn [7] emphasizes the importance of features with significant information in cross-domain translation and considers the relationship between contrastive blocks and surrounding features. Although Qs-Attn [7] achieves feature extraction from both global contextual and local information aspects of images, it fails to account for the details and overall texture of multi-layer features in a single image, resulting in blurred edge features in the generated images. MCL [8] proposes to enhance the discriminator by applying contrastive loss constraint on the output layer features of the discriminator. However, it does not consider the overfitting issue of the generator and embedding blocks.

**Fig 1:**
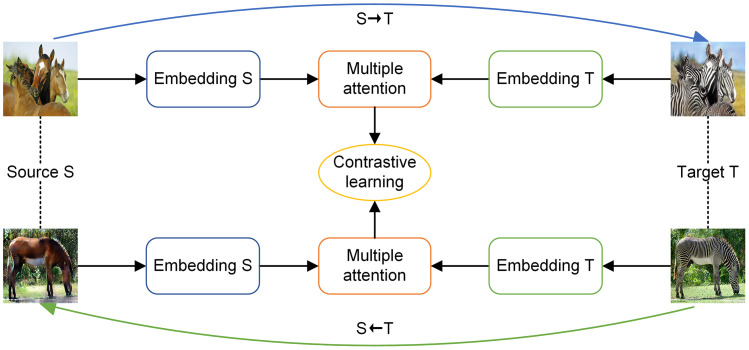
Example of the workflow for the MabCUT method. Train and generate images from both the *S* → *T* and *T* → *S* directions, extract features using independent embedding blocks, and perform contrastive learning by querying key points with the attention extractor.

This paper leverages the advantages of contrastive learning and proposes a fusion multi-attention bidirectional contrastive learning method to enhance the details and textures of the image. The workflow is illustrated in Fig 1. Firstly, MabCUT establishes independent embedding blocks for the source and destination domains. In these blocks, the encoding layer adopts the architecture of depthwise separable convolutions. The models are trained simultaneously from both directions, effectively avoiding overfitting and improving model stability. Then, use pixel-level global and local attention extractors to query the multi-level features of a single image. One way to gauge the significance of traits is via entropy [9]. By calculating the entropy of the attention extractor, important multi-level feature blocks are extracted and the loss is computed through contrastive learning. Simultaneously, depthwise separable convolutions is used in the generator’s architecture to further increase model efficiency and improve the model’s generalization ability.

This article’s primary contributions include the following aspects:

- We propose the MabCUT method for I2IT tasks, which effectively establishes independent embedding blocks based on depthwise separable convolutions for two domains, avoids the problem of mode collapse, and improves the quality of translated images.
- We combine global and local attention extractors to simultaneously query and integrate pixel-level key information from multiple layers of a single image, effectively refining edge features. In addition, we utilize a generator based on depthwise separable convolutions.
- We evaluate our model using three datasets: Horse2Zebra, Cat2Dog, and Cityscapes, against the state-of-the-art techniques currently in use. The results of the trial show that our method produces images with more clarity.

## Related works

### Deep generative models

One kind of generative model that is created by deep learning methods is called a deep generative model. Their primary objective is to learn and generate highly realistic data samples. Deep neural networks are used in these models to simulate the data’s latent distribution and produce new instances based on it. By expanding the dimensions of representation and extrapolating information, generative models can significantly enhance their generation capabilities. Several deep generative models are described below:

Variational Autoencoder (VAE) [10] integrates the concepts of autoencoders and variational inference, allowing it to produce new samples and effectively capture the underlying data distribution. There are two parts of a VAE: an encoder and a decoder. The input data is transformed by the encoder to a latent space containing latent variables. Similarly, the decoder utilizes these latent variables to reconstruct the original data space, producing samples that are almost identical to the original data.

Diffusion models (DMs) [11] comprise two processes: forward and reverse diffusion. The image is gradually subjected to noise during the forward diffusion stage, which ends with a total conversion to random noise. Subsequently, reverse diffusion is applied to gradually eliminate the anticipated noise, thereby generating samples that closely resemble the distribution of real data.

Normalizing Flows (NFs) [12] are employed for modeling complex data distributions. The primary goal of NFs is to establish a mapping from a simple prior distribution, to a more intricate posterior distribution, which represents the target data distribution. This mapping is achieved using invertible transformation functions, enabling the generation of samples that closely resemble the target data.

Generative Adversarial Network (GAN) [13] is composed of a generator and a discriminator. The fundamental principle underlying GAN is adversarial learning, where the discriminator is intended to distinguish between genuine and created images, while the generator aims to produce images that are convincingly realistic. Through an adversarial interplay between the generator and discriminator, they continually enhance each other, ultimately leading to a scenario where the discriminator becomes incapable of distinguishing whether an image is sourced from reality or generated. GAN is a powerful generative model that has found extensive use in applications including super-resolution reconstruction [14], image denoising [15], and image style transfer for image synthesis. However, mode collapse and unstable training plague the original GAN. To address these issues, researchers have proposed various improved GAN models, such as CycleGAN [1], PalGAN [16], and ActFormer [17], to improve the training’s stability and the quality of images that are created.

### Unpaired image-to-image translation

I2IT is often divided into one of two categories: paired image-to-image translation [18] and unpaired image-to-image translation [19]. Paired I2IT, often known as supervised translation, involves establishing a direct correspondence between images in domain *S* and domain *T*. However, obtaining paired images in practical applications is challenging. Consequently, many I2IT tasks are performed in unpaired scenarios. Unpaired I2IT currently encompasses various methods, including cycle consistency [1], shared latent space [20], and the integration of knowledge from different domains to achieve effective translation. At the outset, researchers endeavored to establish a steadfast mapping relationship for unpaired I2IT. To serve this purpose, they introduced the notion of cycle consistency constraint [1]. Concurrently, the UNIT [20] framework put forth the hypothesis of a shared latent space. A matched set of images from different domains may be mapped to a common representation in the latent space, according to this idea. Later, researchers endeavoured to improve the performance of I2IT by integrating methodologies from diverse domains. For instance, InstaFormer [21], Ittr [22] and UVCGAN [23] introduced the transformer architecture into the realm of I2IT, aiming to enhance the quality of generated images.

### Contrastive learning

In unpaired I2IT tasks, finding the mapping link between corresponding areas in different domains is the goal of contrastive learning [7, 24, 25], to connect correlated features, and to impose constraints that maintain vital content throughout the I2IT process. One of the early adopters of contrastive learning in the I2IT work was CUT [4]. It maximizes the mapping relationship between input and output patches through Noise Contrastive Estimation (NCE). Nevertheless, the conventional objective of contrastive learning may lead to confusion as it fails to differentiate between the similarity of negative examples and key points. Instead, contrastive learning treats them on equal grounds by pushing them apart. To address this issue, Large-margin Contrastive Learning was first established by Chen et al. [26] to distinguish intra-cluster and inter-cluster pairings, with the goal of driving the inter-cluster pairs away. None of them have addressed the issue of mode collapse, and they have also not selected meaningful feature blocks during contrastive learning.

## The proposed method

### Bidirectional contrastive learning

Due to the inefficiency of one-way contrastive learning in capturing the mapping information between two domains, our method establishes a separate embedding for each domain based on instance sets *S* = {*s* ∈ *S*} and *T* = {*t* ∈ *T*}. Each embedding block has its own weights, which do not interfere with each other. By using separate encoders and projection layers for the source and target domains, multi-layer features are extracted. Attention matrices are used to select feature blocks that reflect important domain information, maximizing mutual information and achieving high-quality unpaired I2IT tasks.

Contrastive learning primarily aims to establish associations between a query and its relevant positive examples, as well as the irrelevant negative examples. In this process, it maps the query, positive examples, and *N* negative examples to K-dimensional vectors. It is formulated as an (*N* + 1) − *way* classification problem, and the following equation is used to determine the cross-entropy loss:

$$\begin{array}{c} \ell (q,k^{+},k^{-})=-\text{log}\;[\frac{\text{exp}\;(q\cdot k^{+}/\tau )}{\text{exp}\;(q\cdot k^{+}/\tau )+\sum_{n=1}^{N}\text{exp}\;(q\cdot k_{n}^{-}/\tau )}] \end{array}$$ (1)

where *q* represents a crucial point selected from the set *A*(*S*_*s*_), while both *k*^+^ and *k*^−^ are derived from *S*_*s*_. Specifically, *k*^+^ represents a positive example, $k_{n}^{-}$ denotes the n-th negative example, and *τ* signifies a temperature hyperparameter with a specific value of 0.07.

**Fig 2:**
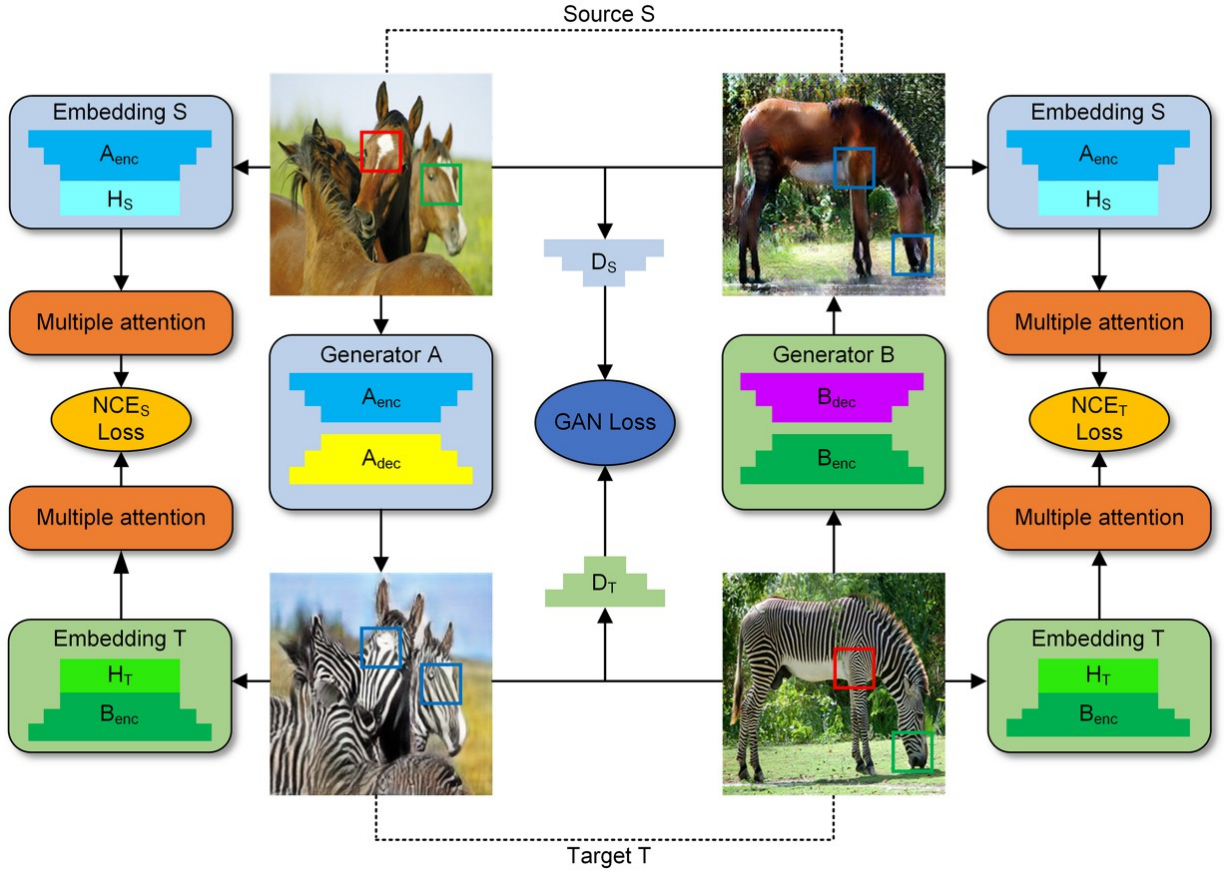
MabCUT framework. The framework achieves bidirectional mapping by utilizing the mappings *A* : *S* → *T* and *B* : *T* → *S*, effectively enabling I2IT between unpaired images while avoiding the strict cycle consistency constraint. In this paper, we define generators *A* and *B*, where *A*_*enc*_ and *B*_*enc*_ serve as encoders, and *A*_*dec*_ and *B*_*dec*_ serve as decoders. By employing *A*_*enc*_ and *H*_*S*_ as the embedding blocks to extract features from various layers of the source domain, and utilizing *B*_*enc*_ and *H*_*T*_ as the embedding blocks for the target domain. The attention matrix selects multiple layers of features through queries and calculates the PatchNCE loss. Additionally, discriminators *D*_*S*_ and *D*_*T*_ compute the GAN loss.

The MabCUT architecture comprises two generators, namely *A* and *B*. Generator *A* is tasked with translating source images into target images, while generator *B* performs the reverse operation. The initial portion of the generator is designated as the encoder, whereas the latter section is referred to as the decoder. Simultaneously, discriminators *D*_*S*_ and *D*_*T*_ are employed to assess the authenticity of the images, utilizing the GAN Loss as the evaluation metric. The comprehensive architecture is depicted in Fig 2. The embedding block *S* incorporates generator A’s encoding layer *A*_*enc*_ and two layers of *MLP*(*H*_*S*_), while the embedding block *T* comprises generator B’s encoding layers *B*_*enc*_ and two layers of *MLP*(*H*_*T*_). By using embedding blocks, multi-layer features are extracted from both the original and goal image. The attention matrix then uses these retrieved features as inputs, which enables the querying and selection of relevant and meaningful feature blocks.

### Attention extractor

In this paper, the attention extractor comprises two main types: global and local. The global attention extractor takes into account the features of all positions in the image, allowing for a comprehensive mapping of the overall image texture. However, it may overlook the correlation between neighboring features. Conversely, the local attention extractor measures the correlation between neighboring features using a fixed-size sliding window, mitigating the limitations of the global attention extractor and improving computational efficiency. Our method employs both global and local attention extractors to combine pixel-level information from multiple layers of a single image. This enables us to capture both the overarching global contextual information and the intricate relationship among neighboring elements.

#### Global attention extractor

**Fig 3:**
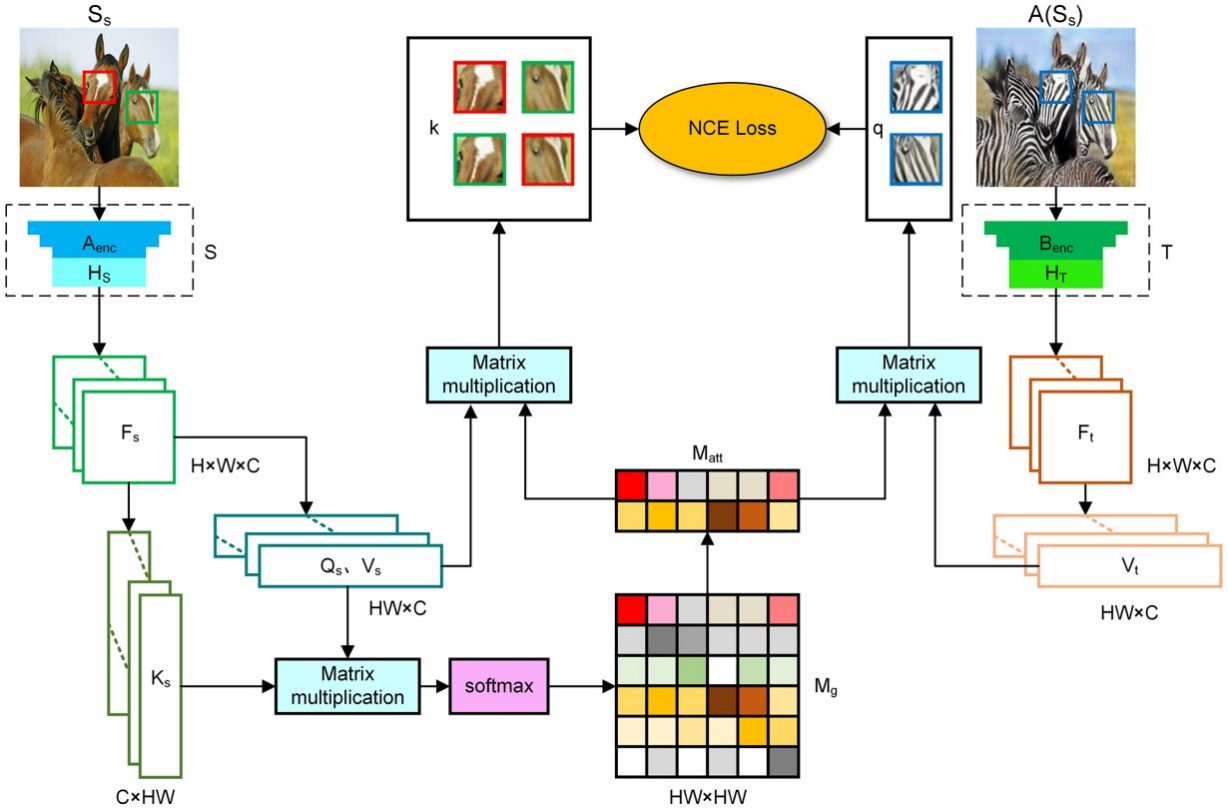
The operational principle of global attention extractor. Image features are extracted from *S*_*s*_ and *A*(*S*_*s*_) using embedding blocks *S* and *T*, respectively. These features are then mapped to three-dimensional matrices *F*_*s*_ and *F*_*t*_. Various operations, including reshaping and transposing, are applied to matrix *F*_*s*_ to derive a two-dimensional attention matrix *M*_*g*_. *N* rows are selected based on the importance of each row in the matrix. These rows are then matched with the value matrices of the target and source domains to find the relevant important points, negative examples, and positive examples. The contrast loss is subsequently calculated. Among them, the feature blocks inside the blue, red, and green boxes represent key points, positive examples, and negative examples respectively.

The workflow of the global attention extractor is illustrated in Fig 3. Initially, we utilize embedding blocks and to derive three-dimensional matrices $F_{s},F_{t}\in R^{H\times W\times C}$. Subsequently, *F*_*s*_ is reshaped into two-dimensional matrices $Q_{s}\in R^{HW\times C}$ and $V_{s}\in R^{HW\times C}$. Simultaneously, the matrix *Q*_*s*_ undergoes a transpose operation, resulting in a new two-dimensional matrix $K_{s}\in R^{C\times HW}$. Then, the matrices *Q*_*s*_ and *K*_*s*_ are multiplied together. By applying the softmax function, each row of the multiplied matrix is activated, leading to the formation of the global attention matrix $M_{g}\in R^{HW\times HW}$. Significant features can be identified by measuring the entropy *H*_*g*_ of each row in *M*_*g*_. The formula is defined as follows:

$$\begin{array}{c} H_{g}\left(i\right)=-\sum_{j=1}^{HW}M_{g}\left(i,j\right)\;\text{log}\;M_{g}\left(i,j\right) \end{array}$$ (2)

where *i* and *j* correspond to the rows and columns of matrix *M*_*g*_, representing the query and the key. As *H*_*g*_(*i*) approaches 0, it indicates that only a few features in row *i* are similar to query *i*. Therefore, as *H*_*g*_(*i*) becomes smaller, the features represented by query *i* are more salient and thus they are more worth retaining. Sort the rows of matrix *M*_*g*_ in ascending order of their entropy values. Select the top *N* rows, resulting in matrix *M*_*att*_.

#### Local attention extractor

Using a square matrix and a stride of 1, with a window size of w×w, the local attention extractor calculates the similarity between each query *i* and its neighboring keys by sliding the window. These calculations result in the value matrix $V_{s}\in R^{HW\times w^{2}\times C}$ and the key matrix $K_{s}\in R^{HW\times w^{2}\times C}$. The reconstructed query matrix $Q_{s}\in R^{HW\times C}$ is multiplied by the key matrix *K*_*s*_, and then activated to get the local attention matrix $M_{l}\in R^{HW\times w^{2}}$ using the softmax algorithm. Additionally, the entropy of each row is calculated. The formula is defined as follows:

$$\begin{array}{c} H_{l}\left(i\right)=-\sum_{j=1}^{w^{2}}\;M_{l}\left(i,j\right)\;\text{log}\;M_{l}\left(i,j\right) \end{array}$$ (3)

where *i* and *j* correspond to the query and key, Sort matrix *M*_*l*_ in ascending order, select the *N* smallest rows, and obtain matrix *M*_*att*_.

The attention matrix *M*_*att*_ is obtained through the use of both global and local attention mechanisms. It is then multiplied, in a routing manner, with the value feature matrices *V*_*s*_ and *V*_*t*_ derived from generated and real images, respectively. This process generates positive examples, negative examples, and key points for the purpose of contrastive learning, which are subsequently employed to calculate the contrastive loss. We extract four layers of features from each embedding block. The first two layers are computed using the global attention extractor, while the last two layers are computed using the local attention extractor.

### Depthwise separable convolutions ResNet

**Fig 4:**
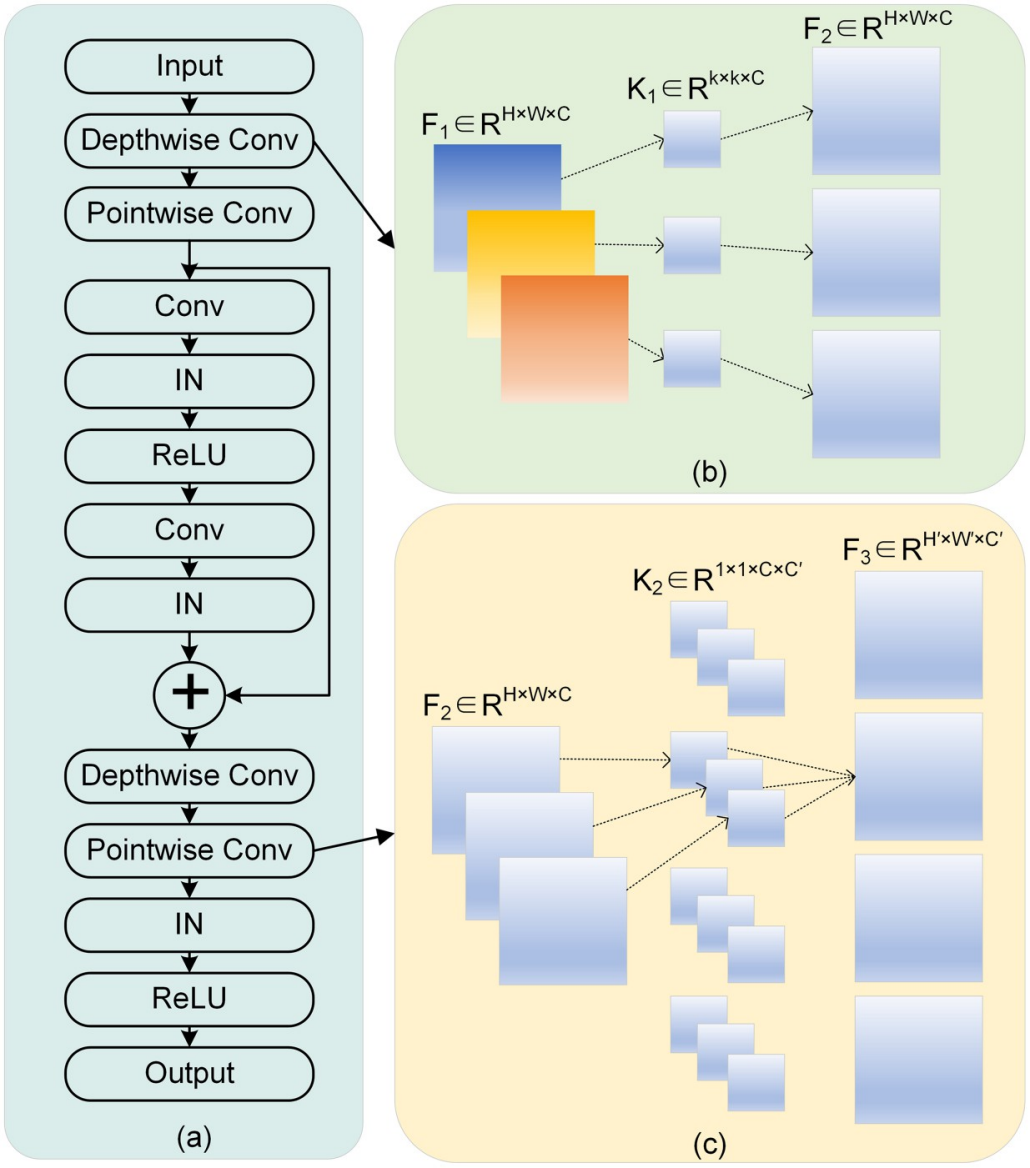
(a) ResNet generator structure. (b) Depthwise convolution. (c) Pointwise convolution.

The workflow of the ResNet generator is illustrated in module (a) of Fig 4. During the upsampling and downsampling process on the feature map, we incorporate depthwise convolution and pointwise convolution. The count of parameters used in model training is decreased by this integration, while also enhancing the handling of local features in the data. Module (b) provides a comprehensive explanation of the operational principles underlying depthwise convolution. It is defined as follows:

$$\begin{array}{c} F_{2}=F_{1}⊗K_{1} \end{array}$$ (4)

where $F_{1}\in R^{H\times W\times C}$ signifies the dimension of input image features. Each channel’s features undergo processing by the convolutional kernel $K_{1}\in R^{k\times k\times C}$, ultimately yielding $F_{2}\in R^{H\times W\times C}$. Subsequently, pointwise convolution is executed, wherein the working principle is exemplified as shown in module (c). This process is established on a 1 × 1 convolution kernel, and it is defined as follows:

$$\begin{array}{c} F_{3}=F_{2}⊗K_{2} \end{array}$$ (5)

where *F*_2_ represents the output of the depthwise convolution, while $K_{2}\in R^{1\times 1\times C\times C^{\prime }}$ represents a 1x1 convolution kernel. The final output is represented as $F_{3}\in R^{H^{\prime }\times W^{\prime }\times C^{\prime }}$.

The kernel size of the conventional convolution technique is denoted by *k* × *k* × *C* × *C*′, and it necessitates a parameter count of *P*_*s*_ = *k*^2^ × *C* × *C*′. In contrast, the parameter count for depthwise separable convolution is represented by *P*_*d*_ = *C* × *C*′ + *k* × *k* × *C* = *C* × (*k*^2^ + *C*′). The ratio between the two is denoted as $\frac{P_{d}}{P_{s}}=\frac{1}{C\prime }+\frac{1}{k^{2}}$. The dimension of the kernel, denoted as *k*, and the number of channels, denoted as *C*′, will both greatly surpass 1 as the network’s depth grows. Consequently, the enhancement in model efficiency becomes increasingly evident.

### Loss function

#### Adversarial loss

The discriminator is continuously updated through backpropagation, while realistic images are generated by the generator, and the differences between the translated and original images are discernible. The discriminator *D*_*T*_ imposes constraints on the translation of images from the domain *T* → *S*. Here is a description of the loss function:

$$\begin{array}{c} L_{dis,t}=E_{t∼T}[\text{log}\;(D_{T}\left(t\right)]+E_{s∼S}[\text{log}\;(1-D_{T}\left(A\left(s\right)\right)] \end{array}$$ (6)

where the discriminator *D*_*T*_ is designed to differentiate between authentic images, denoted as *t*, and translated images, denoted as *A*(*s*). Conversely, the generator *A* is tasked with producing images that are increasingly indistinguishable from real images, thereby enhancing their realism. The discriminator *D*_*S*_ is defined by the following formula:

$$\begin{array}{c} L_{dis,s}=E_{s∼S}[\text{log}\;(D_{S}\left(s\right)]+E_{t∼T}[\text{log}\;(1-D_{S}\left(B\left(t\right)\right)] \end{array}$$ (7)

#### PatchNCE loss

In order to map the image into a feature set ${\left\{z_{l}\right\}}_{L}={\left\{H_{S}^{l}(A_{\text{enc}}^{l}\left(s\right))\right\}}_{L}$, we send the *L* layers that we chose from encoder *A*_*enc*_ to *H*_*S*_. The target domain feature set is represented as ${\left\{{\hat{z}}_{l}\right\}}_{L}={\left\{H_{T}^{l}(B_{\text{enc}}^{l}\left(A\left(s\right)\right))\right\}}_{L}$ in correspondence. The multi-layer features extracted from two embedding blocks are inputted into an attention extractor, facilitating the selection of suitable contrast blocks for each layer of features. By leveraging this approach, we represent the spatial positions as *p* ∈ {1, …, *P*_*l*_} for each layer that has been chosen, the number of spatial locations per layer is denoted by the symbol *P*_*l*_. Every query is associated with a positive example, which is represented as $z_{l}^{p}\in R^{C_{l}}$, whereas all other examples are considered as negative and represented as $z_{l}^{P\backslash p}\in R^{(P_{l}-1)\times C_{l}}$. Furthermore, the notation *C*_*l*_ is used to indicate the count of channels in every layer. Here is how the mapping *A* : *S* → *T* is defined:

$$\begin{array}{c} L_{PatchNCE_{S}}=E_{s∼S}\sum_{l=1}^{L}\sum_{p=1}^{P_{l}}\ell ({\hat{z}}_{l}^{p},z_{l}^{p},z_{l}^{P\backslash p}) \end{array}$$ (8)

The following defines the mapping *B* : *T* → *S*:

$$\begin{array}{c} L_{PatchNCE_{T}}=E_{t∼T}\sum_{l=1}^{L}\sum_{p=1}^{P_{l}}\ell ({\hat{z}}_{l}^{p},z_{l}^{p},z_{l}^{P\backslash p}) \end{array}$$ (9)

#### Identity loss

To retain the essential texture and features of the original image throughout the I2IT process, and to mitigate the occurrence of substantial distortion in the generated images, we introduce the concept of Identity Loss as a constraint. This constraint aims to enhance the authenticity of I2IT. The definition is provided below:

$$\begin{array}{c} L_{identity}\left(A,B\right)=E_{s∼S}[\|B\left(s\right)-s{\|}_{1}]+E_{t∼T}[\|A\left(t\right)-t{\|}_{1}] \end{array}$$ (10)

### General objective

Our primary goal is to accomplish unpaired I2IT with specific features while maintaining the integrity of essential texture and features. To achieve this, the comprehensive loss function of the framework incorporates Adversarial Loss, PatchNCE Loss and Identity Loss. Below are the definitions of these losses:

$$\begin{array}{ccc} L_{loss} & = & \lambda_{GAN}(L_{dis,t}+L_{dis,s})\\ & + & \lambda_{NCE}(L_{PatchNCE_{S}}+L_{PatchNCE_{T}})\\ & + & \lambda_{IDT}L_{identity}\left(A,B\right) \end{array}$$ (11)

In this paper, we establish the hyperparameters λ_*GAN*_ = 1, λ_*NCE*_ = 2, and λ_*IDT*_ = 1, calculate the loss according to the corresponding weights.

## Experiment

### Datasets

The experimental results were assessed on three separate datasets. Within the Horse2Zebra [1] dataset, the images featuring horses serve as the representatives of the source domain set, whereas zebras represent the target domain set. The Cat2Dog [27] dataset consists of 1000 test images and 9892 training images, where the source domain set is composed of cat images and the target domain set is composed of dog images. The Cityscapes [28] dataset is sourced from urban streets, with images from two domains representing German urban street scenes and semantic segmentation labels. The experiment is built upon the three aforementioned datasets. Table 1 displays the quantity of images contained in each dataset. We consider regular horse, semantic segmentation, and cat images as the source domain set, and zebra, street scenes, and dog images as the target domain set.

**Table 1:** Image data for the three datasets.

Dataset	Train		Test	
	Source	Target	Source	Target
Horse2Zebra	1067	1334	120	140
Cat2Dog	5153	4739	500	500
Cityscapes	2975	2975	500	500

### Training details

Our model and all baseline methods were trained on the RTX 3080Ti GPU. The Adam optimizer is used in model training, with the initial learning rate set to 0.0001, parameters set as *β*_1_ = 0.5 and *β*_2_ = 0.999. The model is trained for a total of 400 iterations. However, for the Cat2Dog training set, the Iteration count is explicitly limited at 200. We utilize a PatchGAN [29] discriminator and a depthwise separable ResNet [30] generator, with a batch size of 1. The image dimensions used for testing and training the model are set at 256 × 256. In the case of particular images, we employ a cropping technique from the center to obtain a pixel size of 256 × 256. Similarly, we set the number of rows in the attention extractor to 256 in order to maximize the reflection of the features of the source image.

### Evaluation

#### Metrics

The techniques of Kernel Inception Distance (KID) [31] and Fréchet Inception Distance (FID) [32] are frequently utilized for evaluating the quality of produced images in I2IT tasks. By calculating the distance between features derived from a pretrained image recognition network, the FID [32] measure assesses the similarity between produced images and actual images. The produced and actual images are more alike when the FID [32] value is smaller, serving as an indicator of superior image quality. Using the feature vectors of produced and actual images acquired from the Inception network, the KID [31] computes the squared maximum average difference. The visual resemblance between the produced and actual images is greater when the KID [31] value is smaller. Specifically, the cityscapes dataset employed the pre-trained DRN [33] model for performing segmentation. The correlation between the generated maps and the ground truth maps was assessed, and various evaluation metrics including the mean class accuracy (classAcc), pixel-wise accuracy (pixAcc) and mean average precision (mAP) were computed.

#### Baselines

To ascertain whether the proposed approach is effective in unpaired I2IT, we conducted a comparative analysis of the proposed model using a number of state-of-the-art unsupervised techniques, namely UNSB [34], CycleGAN [1], CUT [4], DCLGAN [35], MCL [8], Qs-Attn [7] and ASGIT [36].

UNSB [34] avoids the curse of dimensionality in unpaired I2IT tasks through regularization and adversarial learning, achieving the transformation between two distributions. Various numbers provide distinct impacts on the model’s NFE values in the range of 1 to 5. We choose the result with the highest image quality for comparison with our model.

CycleGAN [1] employs the concept of cycle consistency to establish an identically between synthesized and actual images. This process entails the incorporation of adversarial loss, cycle consistency loss, and identity loss into the overall loss function.

CUT [4] includes two methods: CUT and FastCUT. CUT uses traditional contrastive learning with the parameter settings λ_*X*_ = 1 and λ_*Y*_ = 1. On the other hand, FastCUT is a faster method that improves computational efficiency by using larger parameters λ_*X*_ = 10 in order to compensate for the absence of identity loss λ_*Y*_ = 0.

DCLGAN [35] proposes two methods, DCLGAN and its variant SimDCL, which splits the target domain’s training from the source domain’s, therefore stabilizing the training process. SimDCL effectively avoids the problem of mode collapse. Similar to our method, DCLGAN has a starting learning rate of *η* = 0.0001 and is trained for 400 epochs. Specifically, SimDCL sets the learning rate to *η* = 0.0002 and is trained for 200 epochs.

MCL [8] utilizes the discriminator’s output layer to the fullest extent possible, using its feature information to compute the contrastive loss. Specifically, the initial learning rate is *η* = 0.0002.

Qs-Attn [7] proposes a query-selection attention module that constrains the preservation of important features during the image translation process, including both the global and local modes.

ASGIT [36] introduces an attention mechanism in the discriminator, generating attention maps for predicted images and transmitting them to the generator. This approach enhances the optimization of the generator.

## Results

### Unpaired I2IT tasks

Through conducting quantitative experiments, we compare the results of our model on the Horse2Zebra, Cat2Dog, and Cityscapes datasets with those of several baseline models. The evaluation of different models is based on the FID [32] and KID [31] metrics. To gain a more intuitive understanding of the model’s performance on different datasets, we conduct separate comparisons of the three result sets. This allows us to analyze the strengths and weaknesses of our model in comparison to other baseline models.

**Fig 5:**
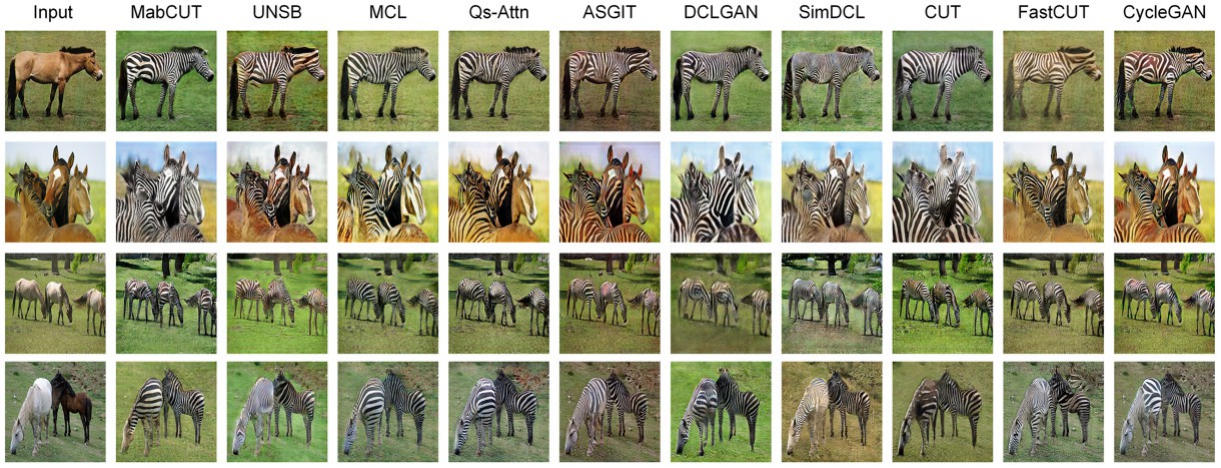
Comparison results on the Horse2Zebra dataset.

The results depicted in Fig 5 indicate that other models display distortion when translating from horses to zebras, causing the zebra’s details to become blurred and the textures to appear unnatural. In contrast, our model-generated images not only retain the characteristics of the horses present in the source images but also exhibit a broader coverage of zebra stripes and smoother textures. The quantitative experimental results shown in Table 2 show that our model performs best in both metrics, boasting a significant 2.7 points lead over the MCL [8] model, which holds second place in terms of FID [32].

**Table 2:** FID and KID×100 scores on the Horse2Zebra dataset, with the best performance indicated in bold.

Method	Horse→Zebra	
	FID↓	KID×100↓
CycleGAN	78.3	2.1052±0.1168
FastCUT	73.1	2.0289±2.0918
CUT	45.7	0.5199±0.0008
DCLGAN	43.3	0.5443±0.0499
SimDCL	47.4	0.5961±0.0278
ASGIT	65.0	1.3126±0.0493
Qs-Attn	43.1	0.4916±0.0549
MCL	42.9	0.5558±0.0502
UNSB	53.9	0.7757±0.0899
MabCUT(Ours)	**40.2**	**0.4890±0.0646**

**Fig 6:**
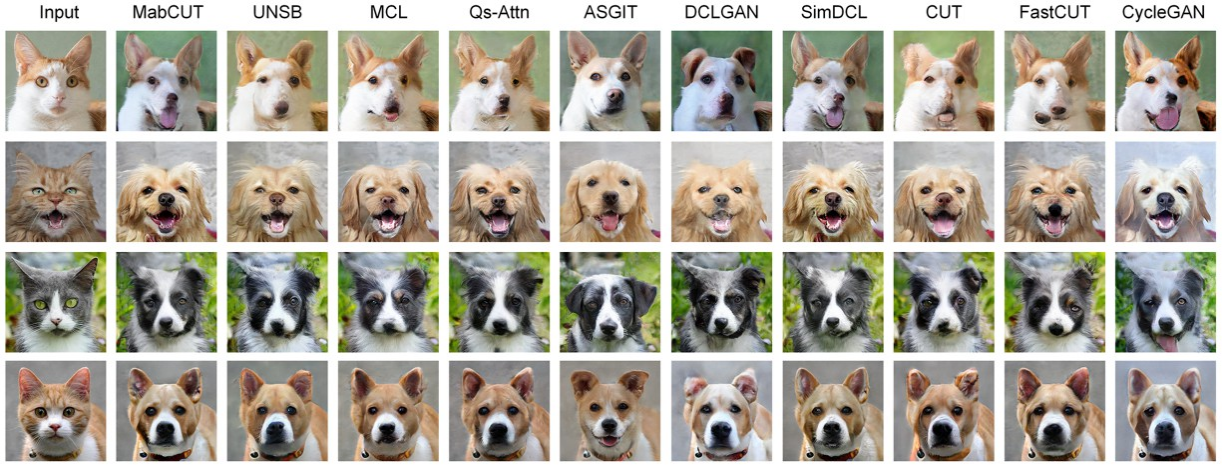
Comparison results on the Cat2Dog dataset.

Fig 6 provides an objective assessment of the performance exhibited by various models in the task of translating Cat2Dog images. Through qualitative comparison, it has been observed that, while Qs-Attn [7], MCL [8], DCLGAN [35], and CUT [4] are capable of generating more vivid facial features compared to other baseline methods, they still exhibit deficiencies in terms of visual effects when compared to our model. Specifically, the dog images generated by these models lack smoothness. The two scores presented in Table 3 serve to reinforce our analysis of the performance displayed by various advanced models. Our approach achieved scores of 59.8 and 2.2698 for FID [32] and KID [31], respectively, outperforming all baseline methods.

**Table 3:** FID and KID×100 scores on the Cat2Dog dataset.

Method	Cat→Dog	
	FID↓	KID×100↓
CycleGAN	85.2	3.6241±0.4371
FastCUT	92.3	5.2242±0.5268
CUT	75.0	3.6043±0.3985
DCLGAN	60.4	2.4148±0.3903
SimDCL	65.8	2.6486±0.3733
ASGIT	74.2	3.4032±0.4906
Qs-Attn	73.0	3.4610±0.4390
MCL	71.6	3.2903±0.4317
UNSB	73.8	3.3984±0.4848
MabCUT(Ours)	**59.8**	**2.2698±0.2601**

**Fig 7:**
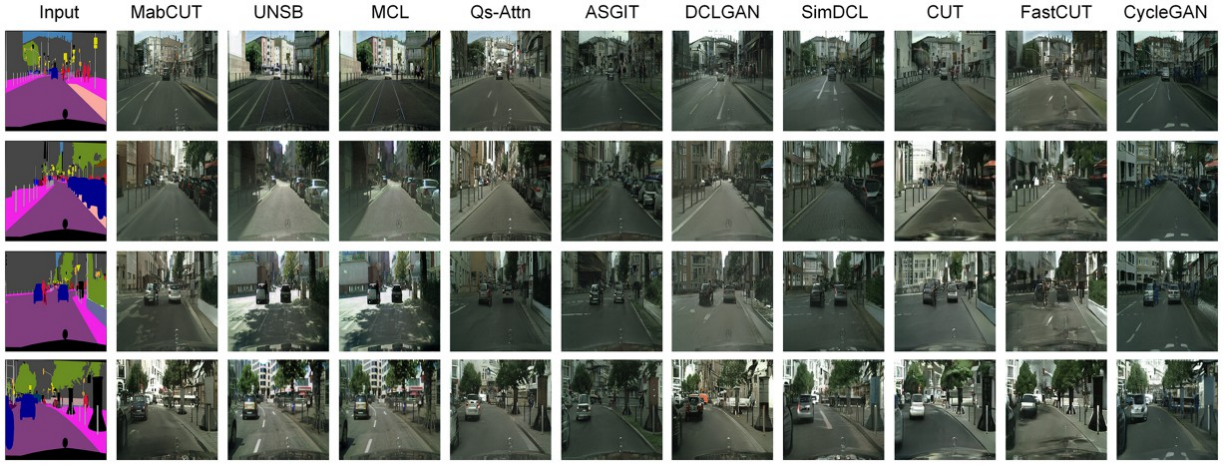
Comparison results on the Cityscapes dataset.

From Fig 7, it is not difficult to observe that our model can produce realistic content based on semantic labels, including cars, pedestrians, houses, and trees, among others. In contrast, models such as DCLGAN [35] and CUT [4] produce indistinct content that fails to accurately depict the information in the target domain. Our model exhibits significant advantages in generating street scenes, as demonstrated in Table 4. Our model outperforms other methods in terms of classAcc, mAP, KID [31], and FID [32] metrics, but slightly underperforms compared to Qs-Attn [7] in the pixAcc metric. However, upon observing Fig 7, it becomes evident that the image realism of our model surpasses that of Qs-Attn [7].

**Table 4:** FID and KID×100 scores on the Cityscapes dataset.

Method	Cityscapes				
	mAP↑	pixAcc↑	classAcc↑	FID↓	KID×100↓
CycleGAN	20.8	56.3	25.6	73.5	3.1327±0.5732
FastCUT	18.9	68.2	30.1	69.6	2.7319±0.6758
CUT	24.5	68.8	30.6	56.7	1.7479±0.5873
DCLGAN	23.7	75.4	28.9	55.6	1.4645±0.2306
SimDCL	21.1	69.9	21.4	58.9	1.9696±0.2566
ASGIT	22.3	71.2	26.8	65.0	2.7608±1.2482
Qs-Attn	27.1	**80.4**	31.7	51.3	1.4792±0.5776
MCL	26.4	77.3	28.6	50.7	1.4236±0.1656
UNSB	25.8	76.7	27.3	53.4	1.6537±0.1713
MabCUT(Ours)	**28.2**	78.9	**34.1**	**46.7**	**0.6557±0.1461**

In this paper, we conducted quantitative comparative experiments and displayed qualitative results to compare our model with nine advanced baseline models. Our comparisons were based on factors such as texture, visual effects, clarity, and details. We analyzed the advantages of our model from various perspectives in contrast to the other models.

### Ablation study

Our method demonstrates superior translation performance when compared to all baseline models. To delve deeper into the influence of each contribution on the efficacy of our model, we conducted ablation experiments. These experiments were based on the three datasets utilized in the aforementioned experiments, enabling comprehensive ablation comparisons.

**Fig 8:**
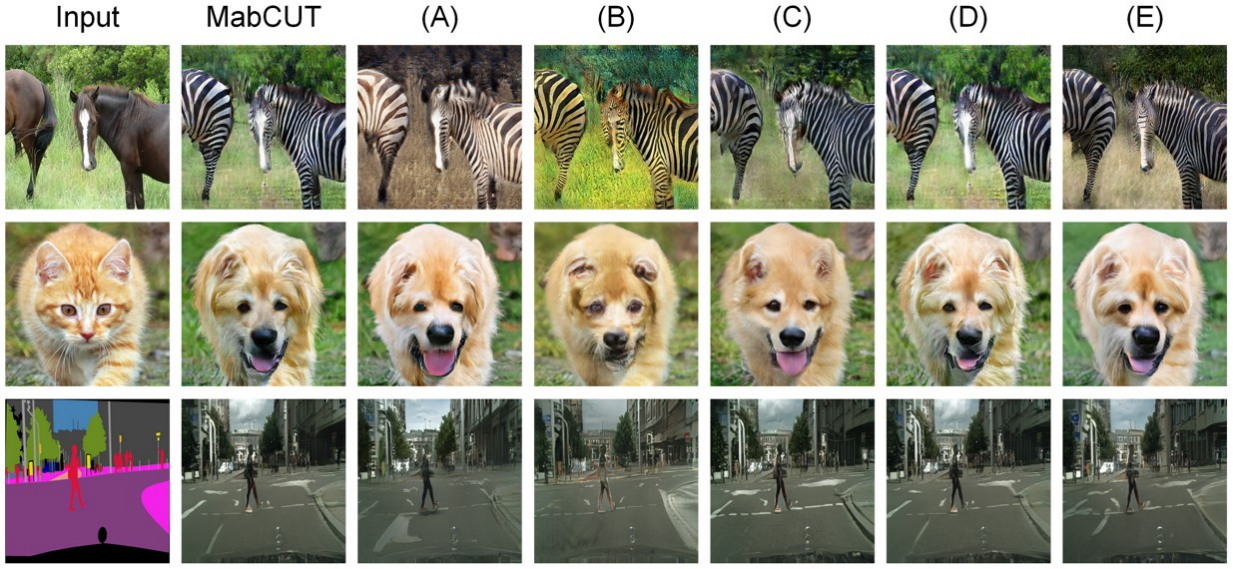
Qualitative ablation experiment. Here, MabCUT represents the results of this model, and (A)→(E) are the I2IT results of each ablation module in sequence.

Our model incorporates a bidirectional contrastive learning mechanism and integrates a multiple attention extractor method into the feature extraction process. Consequently, we establish the following comparisons: (A) Using unidirectional contrastive learning and applying an identical embedding block. (B) Eliminating the attention extractor and employing randomized selection of contrast blocks. (C) All feature layers are computed using the global attention extractor. (D) All feature layers are computed using the local attention extractor. (E) The generator and embedding blocks eliminate depthwise separable convolutions.

(A) Through an investigation into the effects of unidirectional contrastive learning methods, we employ a unilateral attention extractor to extract feature blocks. The results depicted in Fig 8 indicate a substantial degradation in performance on the Horse2Zebra dataset. Additionally, the quantitative findings presented in Table 5 demonstrate that MabCUT has improved by 28.6%, 6%, and 12.4% respectively compared to the unidirectional comparative learning method across three datasets. This proves the effectiveness of MabCUT in establishing independent embedding blocks for different domains.
(B) In order to examine the effectiveness of actively selecting meaningful keypoints, we adopted the strategy of randomly selecting contrastive blocks from the CUT [4] framework in our experimental setup. Analysis of the results in Table 5 reveals higher overall scores across all three datasets. More specifically, the scores for Cat2Dog and Cityscapes are recorded as 74.6 and 57.1, respectively. Interestingly, among all the ablation contrastive models, this approach demonstrates the lowest performance, indicating that MabCUT’s attention matrix method is capable of learning superior contrastive loss and achieving stable training.
(C) Use the global attention extractor to query and select key points from all feature layers. The global attention extractor focuses more on the overall features of the image. When observing Fig 8, it becomes evident that the translated visual quality of the image is relatively low, and Table 5 demonstrates that the scores are worse than those of MabCUT across different datasets, indicating that the global attention extractor pays less attention to neighboring features.
(D) Query and select key points from all feature layers using the local attention extractor. The model scores 46.9 on the Cityscapes dataset, which is close to the score of the MabCUT model. In contrast, it significantly underperforms the MabCUT model in the other two datasets, highlighting the efficacy of the MabCUT model’s approach to combining local and global attention. The correlations between adjacent features and the general texture of the picture may both be captured by MabCUT model.
(E) The depthwise separable convolutions were removed from the generator and embedding blocks, leading to inferior experimental results compared to MabCUT. The analysis of Table 5 reveals a noticeable increase in the FID scores for the Horse2Zebra and Cat2Dog datasets, with respective increments of 9.4 and 7.6. These findings suggest a deterioration in the overall quality of the images. Moreover, the parameter count increased to 29.274M, surpassing MabCUT’s 28.508M. This observation suggests that the utilization of depthwise separable convolutions not only reduces parameter count and enhances computational efficiency but also effectively captures data features, thereby improving the model’s generalization ability.

**Table 5:** The quantitative comparison results from ablation experiments. In order to demonstrate the effects of each of our contributions on I2IT.

Method	Horse→Zebra	Cat→Dog	Cityscapes
	FID↓	FID↓	FID↓
(A)	56.3	63.6	53.3
(B)	53.0	74.6	57.1
(C)	45.1	63.2	48.1
(D)	43.3	67.1	46.9
(E)	49.6	67.4	49.5
MabCUT	**40.2**	**59.8**	**46.7**

### User study

**Fig 9:**
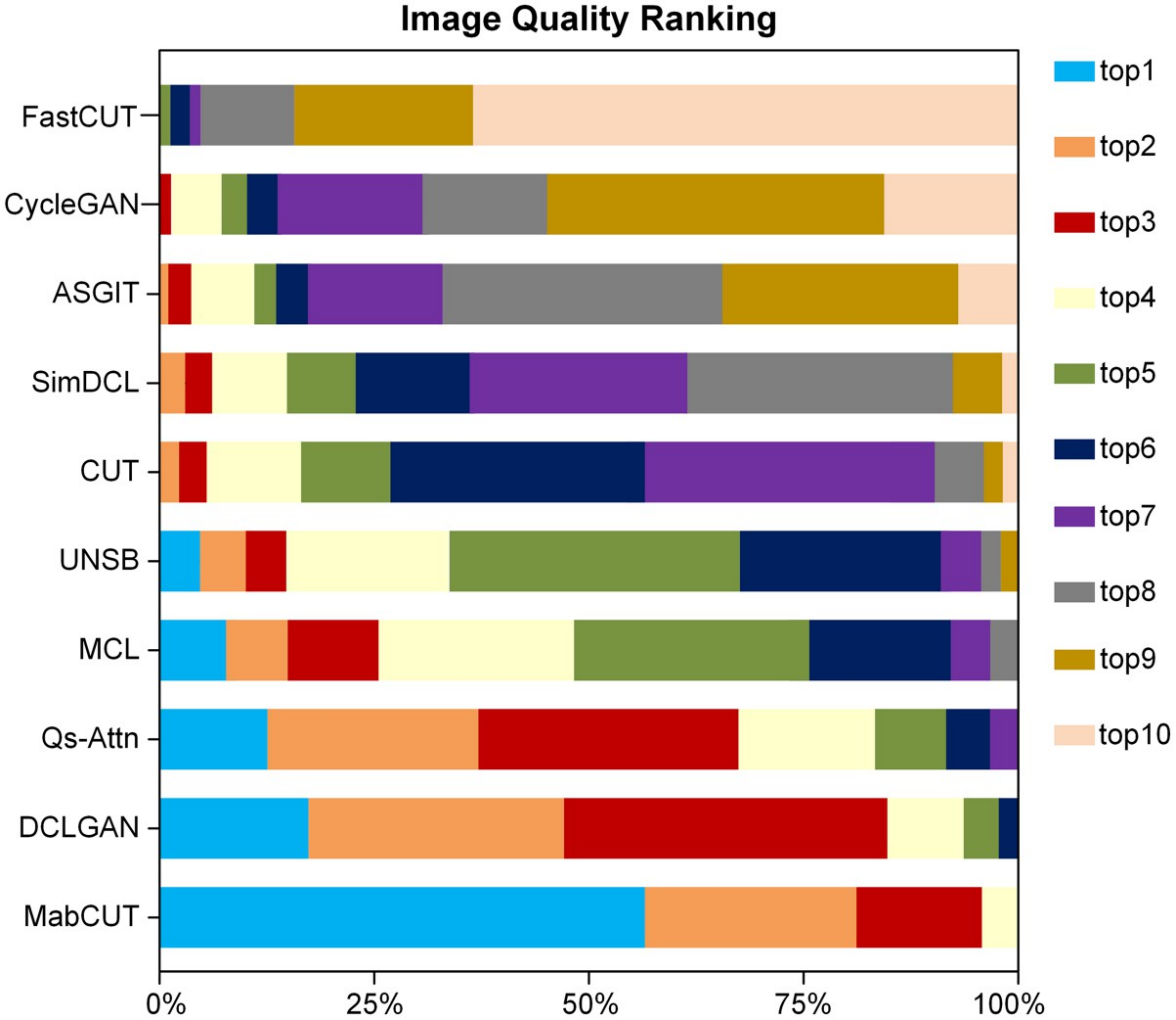
User study results. In this paper, we consolidate and compute the proportional rankings provided by users across various models. Subsequently, we conduct a thorough analysis of the quality of these models using detailed graphical representations. The horizontal axis shows the percentage of ranks, while the vertical axis refers to the various models.

To substantiate our model’s efficacy in unpaired I2IT, we recruited 30 volunteers based on visual perception and compared MabCUT with other baseline models on three datasets. From each dataset, we chose 20 images at random. Volunteers were asked to evaluate the quality of image translation from a visual perception perspective and rank the translation results of different models. As shown in Fig 9, MabCUT exhibited significant quality advantages compared to the baseline models, ranking first in 58% of user evaluations.

## Conclusions and discussion

In this paper, we propose a bidirectional contrastive learning method based on multiple attention extractor. This method extracts features from both the source and target domains separately using independent embedding blocks, and integrates both global and local attention extractor to identify important feature blocks for contrastive learning. At the same time, while ensuring the quality of image translation, we incorporate depthwise separable convolutions into the generator to reduce training costs. Through comprehensive comparison experiments, ablation experiments, and user studies, we have validated the effectiveness of the MabCUT method in the field of unpaired I2IT. However, our model changes the background color of the image during the process of I2IT, which affects the translation results. The problem of background preservation during the I2IT process should be the main focus of future study.
